# Human Immunodeficiency Virus Type 2 (HIV-2) Gag Is Trafficked in an AP-3 and AP-5 Dependent Manner

**DOI:** 10.1371/journal.pone.0158941

**Published:** 2016-07-08

**Authors:** Justine E. Alford, Michela Marongiu, Gemma L. Watkins, Emma C. Anderson

**Affiliations:** School of Life Sciences, University of Warwick, Coventry, United Kingdom; Lady Davis Institute for Medical Research, CANADA

## Abstract

Although human immunodeficiency virus (HIV) types 1 and 2 are closely related lentiviruses with similar replication cycles, HIV-2 infection is associated with slower progression to AIDS, a higher proportion of long term non-progressors, and lower rates of transmission than HIV-1, likely as a consequence of a lower viral load during HIV-2 infection. A mechanistic explanation for the differential viral load remains unclear but knowledge of differences in particle production between HIV-1 and HIV-2 may help to shed light on this issue. In contrast to HIV-1, little is known about the assembly of HIV-2 particles, and the trafficking of HIV-2 Gag, the structural component of the virus, within cells. We have established that HIV-2 Gag accumulates in intracellular CD63 positive compartments, from which it may be delivered or recycled to the cell surface, or degraded. HIV-2 particle release was dependent on the adaptor protein complex AP-3 and the newly identified AP-5 complex, but much less so on AP-1. In contrast, HIV-1 particle release required AP-1 and AP-3, but not AP-5. AP-2, an essential component of clathrin-mediated endocytosis, which was previously shown to be inhibitory to HIV-1 particle release, had no effect on HIV-2. The differential requirement for adaptor protein complexes confirmed that HIV-1 and HIV-2 Gag have distinct cellular trafficking pathways, and that HIV-2 particles may be more susceptible to degradation prior to release.

## Introduction

Human immunodeficiency virus (HIV) types 1 and 2 are closely related lentiviruses that are the causative agents of Acquired Immune Deficiency Syndrome (AIDS). If left untreated, the majority of patients infected with HIV-1 will develop AIDS within 8–10 years of infection, whereas only a minority of patients infected with HIV-2 will develop AIDS, and over a longer timespan [1, 2]. HIV-2 is also less transmissible than HIV-1 [3], and has not spread widely from West Africa. These differences are likely a consequence of the different levels of virus found in patients during the clinically asymptomatic phase of HIV-1 or -2 infection [4]. Cohort studies have shown that circulating viral loads of HIV-2 are lower than HIV-1 during infection, despite similar levels of integrated proviral DNA [5], although the reasons for this difference remain unclear. The replication cycles of the two lentiviruses are similar, but HIV-2 is less well-studied than HIV-1, leaving potential mechanistic differences yet to be characterized. Understanding how HIV-1 and HIV-2 differ in the production and release of viral particles could enable the discovery of new ways to target the more pathogenic HIV-1, and more specific ways to target HIV-2.

The assembly and budding of lentiviral particles is directed by the Gag polyprotein; expression of HIV-1 or -2 Gag alone can result in the production of virus-like particles that bud out of cells [6, 7]. During viral infection, Gag and GagPol polyproteins are synthesized in the cytoplasm before assembling into multimeric structures at membranes. The major site of HIV-1 particle assembly in T cells and a number of cell lines such as 293T and HeLa is the plasma membrane [8, 9], in cholesterol and tetraspanin-enriched microdomains [10]. However the route by which HIV-1 Gag arrives at the plasma membrane is less clear; trafficking via endosomal compartments to the plasma membrane has been shown [11], as has direct trafficking to the plasma membrane followed by internalization into endosomes [9]. In macrophages, HIV-1 assembles at intracellular membranes; originally described as late endosomes or multivesicular bodies [12, 13], these virion containing compartments (VCCs) have been shown to be non-acidic [14], tetraspanin-enriched [15], and linked by very narrow channels to the plasma membrane [14].

The trafficking of HIV-1 Gag from its site of synthesis to its site(s) of assembly may be a passive, diffusion-limited process, or an active process mediated, for example, by cellular vesicular transport pathways. Supporting this, the clathrin adaptor proteins AP-1, -2 and -3 have been shown to interact with HIV-1 Gag and affect HIV-1 particle release [16–18]. Clathrin adaptor proteins are involved in the vesicular trafficking of proteins between the *trans*-Golgi network, endosomes, lysosomes and plasma membrane. The adaptor proteins recognize motifs on membrane-bound cargo proteins and promote the formation of clathrin coated cargo vesicles. AP-2 is found at the plasma membrane and is involved in clathrin-mediated endocytosis, AP-1 is found on Golgi and endosomal membranes, and AP-3 on endosomal and lysosomal membranes [19]. A new adaptor protein (AP-5) has recently been discovered [20] and is found on late endosomal/lysosomal membranes [21], although much less is known about the function of this protein.

In this study we investigated the distribution of HIV-2 Gag in HeLa and 293T cells and the role of AP-1, -2, -3 and -5 in HIV-2 Gag trafficking. We found that HIV-2 Gag traffics to intracellular CD63 positive compartments. HIV-2 particle release was dependent on AP-3, which was recruited to Gag-containing compartments, and AP-5, but much less so on AP-1. In contrast, HIV-1 particle release required AP-1 and AP-3, as previously shown, but not AP-5. AP-2, which has previously been shown to inhibit HIV-1 particle release, had no effect on HIV-2 release.

## Materials and Methods

### Plasmids and siRNAs

Proviral plasmids used in this study were pSVC21ΔBgl (HIV-1 HxB2 strain with nucleotides 6587–7167 deleted), pSVR (HIV-2 ROD strain) and pSVRΔNB [22]. pSVR is a full length HIV-2 molecular clone, pSVC21ΔBgl and pSVRΔNB have deletions in the env gene but contain a functional Rev response element. These plasmids were a gift from Professor Andrew Lever. Custom siRNAs based on published siRNA sequences were ordered from Life Technologies: AP-1γ GCGCCUGUACAAAGCAAUU [16]; AP-2μ GUGGAUGCCUUUCGGGUCA [23]; AP-3δ CCCUGUCCUUCAUUGCCAA [16]. AP-5μ ON-TARGETplus AP5M1 J-015523-09 [20] was ordered from Dharmacon. *Silencer* negative control #2 was also used (Life Technologies). All siRNAs were used at 10 nM final concentration except AP-5μ siRNA which was used at 25nM, as previously published [20].

### Tissue culture and transfections

HeLa (ATCC: CCL-2) and human embryonic kidney 293T (ATCC: CRL-11268) cells were maintained in Dulbecco’s modified Eagle’s medium supplemented with 10% fetal calf serum. Transfections were carried out using Lipofectamine 2000 (Invitrogen) in 12 well plates using 0.5 μg / well proviral plasmid, according to the manufacturer’s instructions. For western blotting, cells were harvested 42 hours post-transfection using passive lysis buffer (Promega). Total protein content of each cell sample was measured using the BCA protein assay kit (Thermo Scientific). Cell supernatants were filtered through a 0.2 μm membrane and virions precipitated overnight at 4°C with 0.5 vol 30% PEG-8000 in 0.4 M NaCl. Viral particles were pelleted and resuspended in PBS. Following SDS polyacrylamide gel electrophoresis and electroblotting to nitrocellulose, viral Gag proteins were detected using polyclonal antiserum to HIV-1 p24 (ARP432, Dr G Reid) or polyclonal antiserum to SIV p27 (ARP414, Dr M Page), from Programme EVA, Centre for AIDS Reagents, NIBSC. Western blots were analyzed using Image J.

### Confocal immunofluorescence microscopy

Cells were plated and transfected with siRNAs and/or proviral plasmids on cover slips in 12 well plates. 42 hours post-transfection, cells were fixed in PBS / 10% formaldehyde for 10 minutes and permeabilized in PBS / 0.5% NP40 for 10 minutes. Cells were then blocked in PBS / 1% BSA for 1 hour and stained using rabbit antiserum to HIV-1 p24 (ARP432) or sheep antiserum to SIV p27 (ARP414), followed by donkey Alexa Fluor 594-tagged anti-rabbit or anti-sheep IgG (Invitrogen). Other antibodies used were mouse monoclonal antibodies against CD63 (Abcam ab8219), CD81 (Santa Cruz sc23962), AP-3δ (BD Biosciences 611328) and clathrin heavy chain (X22, a gift from Professor Frances Brodsky). These were then detected with donkey Alexa Fluor 488-tagged anti-mouse IgG (Invitrogen). Cover slips were mounted onto standard glass microscope slides using VectaShield (Vector Laboratories) with DAPI and visualized using a 63x oil objective (Leica SP5). Co-localization analysis was carried out using Image J.

### Reverse transcription-quantitative PCR (RT-qPCR)

HeLa cells were harvested 42 hours post-transfection with AP-5 and negative control siRNAs. RNA was isolated with an RNeasy kit (Qiagen) and cDNA was synthesized using a high capacity RNA-to-cDNA kit (Applied Biosystems). The cDNA was used as a template for real time PCR with 2x Fast SYBR Green master mix (Applied Biosystems) and the following primers: AP5f CACCGTGAGATTCTCCAGACG and AP5r TGGCCAGAGTTCTTCACCTCC; gapdhf TCTCCTCTGACTTCAACAGCGAC and gapdhr CCCTGTTGCTGTAGCCAAATTC. Amplification and detection were carried out using an ABI 7000 Sequence Detection System.

## Results

### HIV-2 Gag localizes to intracellular compartments

**Fig 1:**
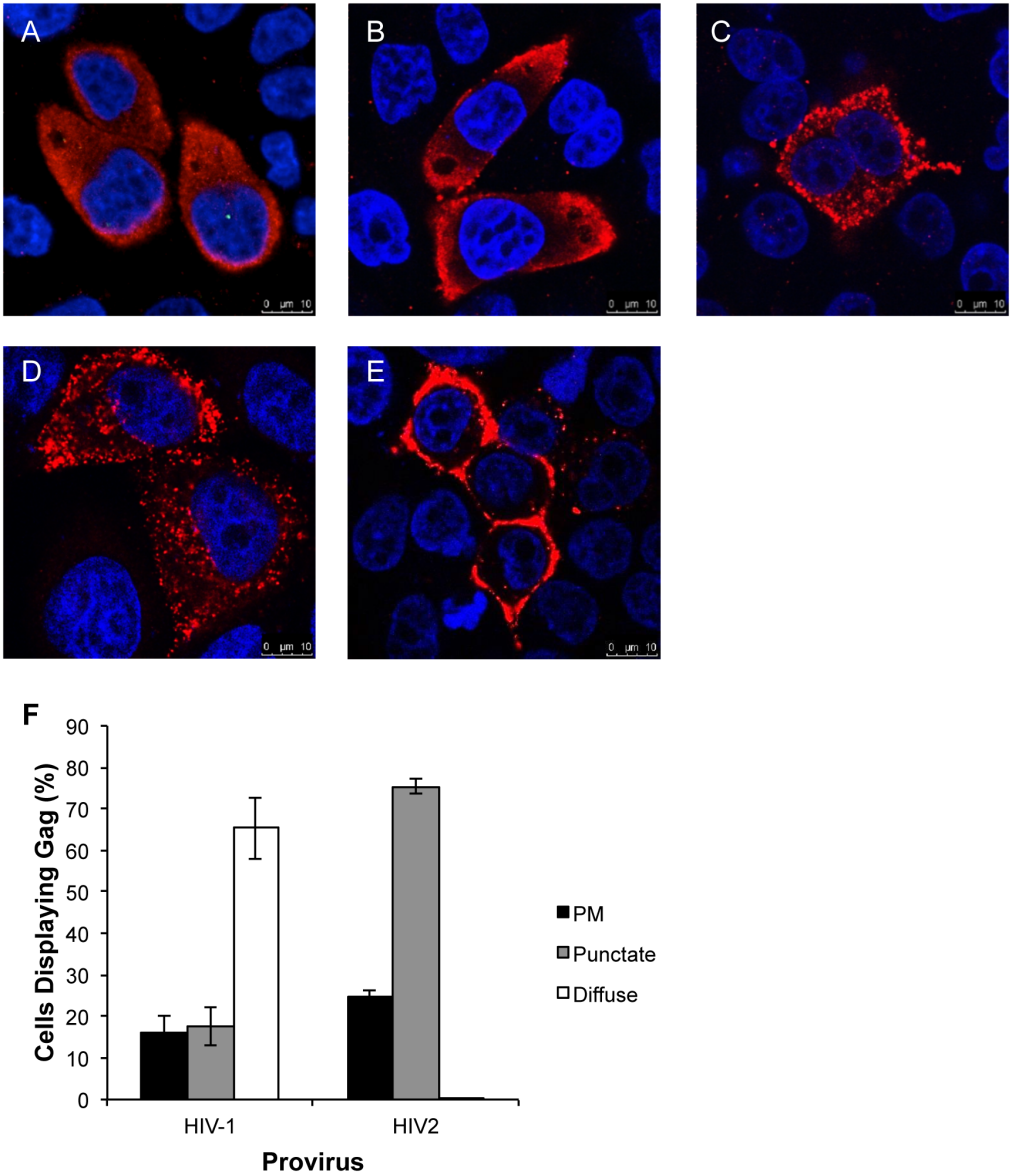
HIV-1 and -2 Gag differ in their subcellular distribution. Confocal immunofluorescence microscopy of HeLa cells transfected with envelope-deleted proviral plasmids. Gag immunofluorescence is shown in red, DAPI staining in blue. (A-C) HIV-1, (D, E) HIV-2. Scale bars are shown in the bottom right corner of each image. (F) Quantification of Gag distribution is shown graphically. Several hundred cells from three independent experiments were assessed by two different people. Cells were scored as having predominantly plasma membrane (PM), punctate intracellular (punctate), or diffuse cytoplasmic (diffuse) distribution of Gag. Error bars represent the standard deviation.

HIV-1 Gag has been shown to aggregate and assemble into viral particles at the plasma membrane in T cells, 293T cells and HeLa cells [8, 9], whereas in macrophages intracellular VCCs have been shown to be the site of assembly [14, 15]. We investigated the cellular distribution of HIV-1 and -2 Gag using confocal immunofluorescence microscopy of cells transfected with proviral plasmids. HIV-1 Gag was observed to display one of three distributions: diffuse in the cytoplasm (Fig 1A), plasma membrane-associated (Fig 1B), or punctate (Fig 1C), as has been previously reported [24]. HIV-2 Gag was observed to be punctate (Fig 1D) or plasma membrane-associated (Fig 1E). The different patterns of HIV-1 and HIV-2 Gag distribution were quantified by assessing several hundred cells from independent experiments. For HIV-1, around 65% of cells showed a predominantly diffuse distribution of Gag, 18% punctate and 16% plasma membrane-associated, whereas for HIV-2, no cells had a diffuse distribution, 75% showed a punctate distribution, and 25% of cells had plasma membrane-associated Gag (Fig 1F).

**Fig 2:**
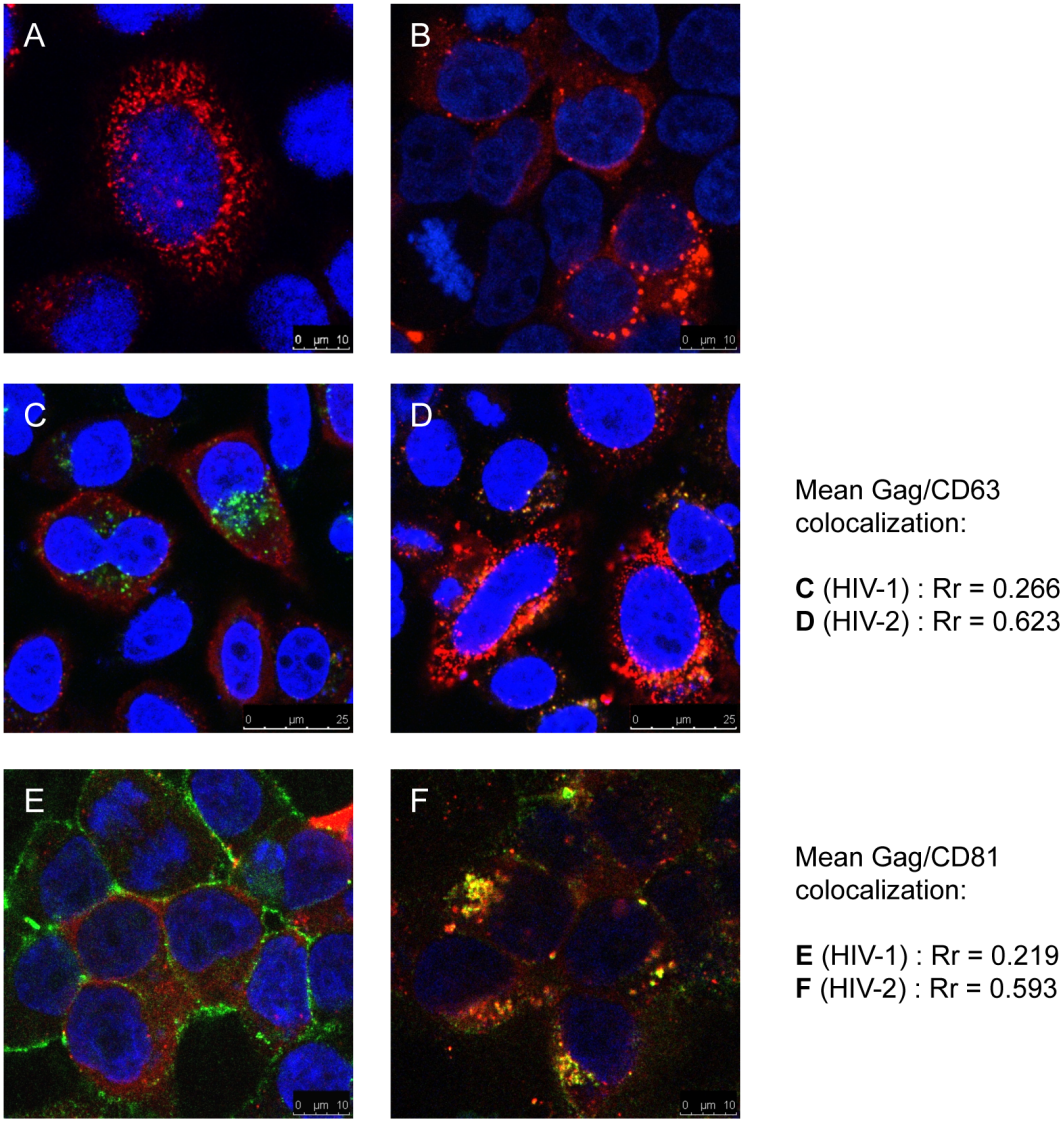
HIV-2 Gag localizes to CD63+/CD81+ compartments. Confocal immunofluorescence microscopy of HeLa cells (unless stated) transfected with proviral plasmids. Gag immunofluorescence is shown in red, DAPI staining in blue. (A) full length HIV-2 (expressing Env), (B) HIV-2 in 293T cells. (C) HIV-1, co-staining for Gag (red) and CD63 (green), (D) HIV-2, co-staining for Gag (red) and CD63 (green), (E) HIV-1, co-staining for Gag (red) and CD81 (green), (F) HIV-2, co-staining for Gag (red) and CD81 (green). Mean Gag and CD63 or CD81 co-localization (Pearson’s correlation coefficient, Rr) is shown to the right of images C and D or E and F respectively. Scale bars are shown in the bottom right corner of each image.

The predominantly punctate distribution of HIV-2 Gag was observed in both the presence (Fig 2A) and absence (Fig 1) of HIV-2 Env, indicating that Env does not affect HIV-2 Gag localization. HIV-2 Gag also had a punctate distribution in 293T cells (Fig 2B), which indicates that it is not a pattern that is specific to HeLa cells or due to tetherin expression [25]. To investigate the identity of the intracellular compartment to which HIV-2 Gag is associated, co-localization studies were carried out with the tetraspanins CD63 (late endosome marker) and CD81, a plasma membrane protein that has been shown to be present in macrophage VCCs [15]. HIV-1 Gag did not co-localize with either CD63 (Fig 2C) or CD81 (Fig 2E), with a Pearson’s correlation coefficient (Rr) of 0.266 and 0.219 respectively. However, HIV-2 Gag co-localized strongly with both CD63 (Fig 2D, Rr = 0.623) and CD81 (Fig 2F, Rr = 0.593). Notably, in cells expressing HIV-2 Gag, CD81 showed a punctate intracellular distribution in addition to the plasma membrane association seen in cells expressing HIV-1 Gag. This suggests that either these intracellular compartments are linked via microchannels to the plasma membrane, or that CD81 has been internalized from the cell surface.

### The effect of depleting adaptor proteins 1, 2 or 3 shows different trafficking pathways of HIV-1 and HIV-2 Gag

**Fig 3:**
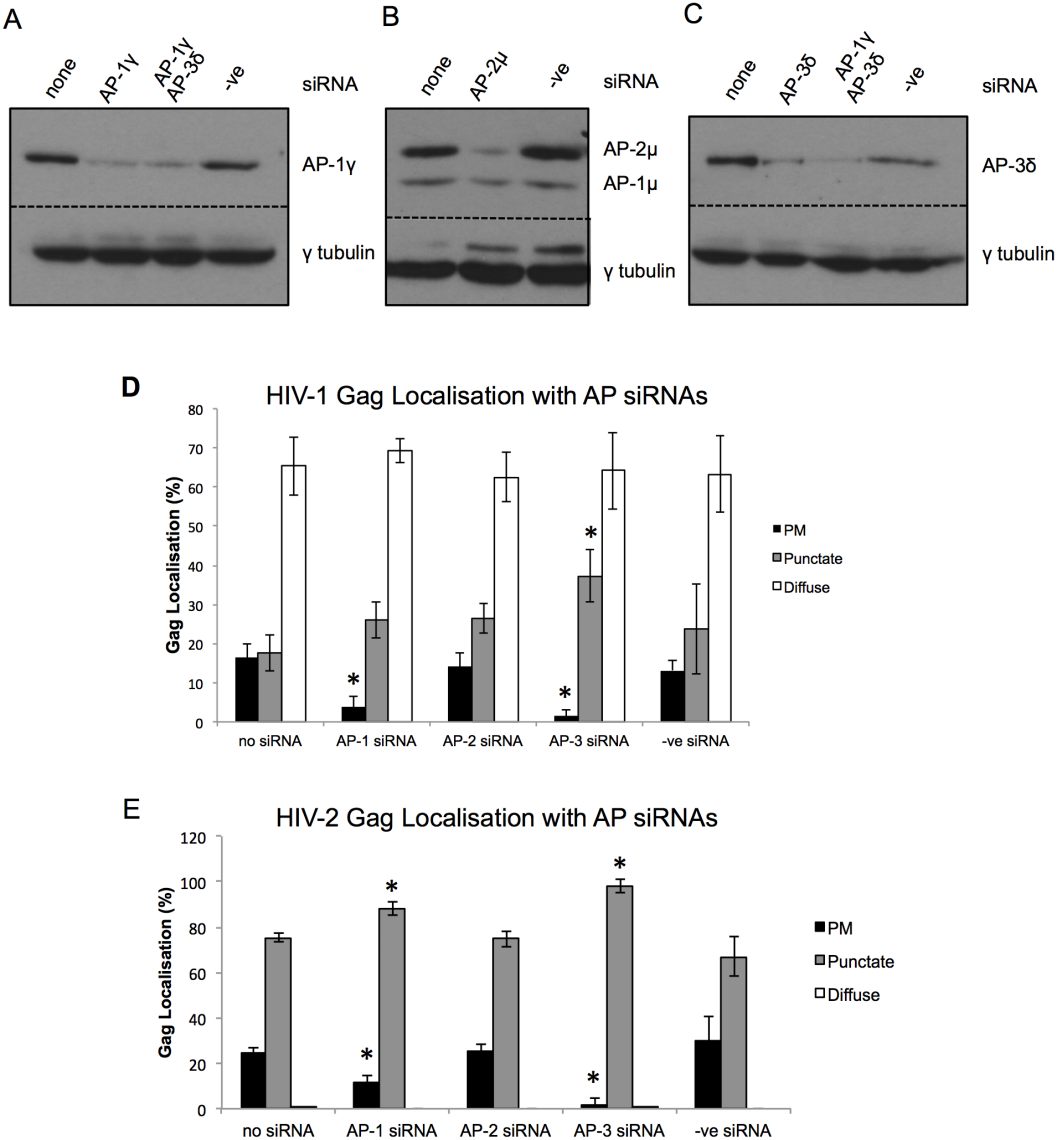
siRNA-mediated knockdown of AP-1 or AP-3 reduces plasma membrane localization and increases punctate distribution of HIV-2 Gag. (A-C) HeLa cells were harvested 42 hours post-transfection with 10 nM siRNA against AP-1γ, AP-2μ, AP-3δ, AP-1γ and AP-3δ, or negative control siRNA (-ve), or mock transfected (none). Western blots of cell lysates were probed with antibodies against AP-1γ (A), AP-2μ (B) and AP-3δ (C). The blots were also probed with an antibody against γ tubulin, as a control. In (B), the antibody that recognizes AP-2μ also recognizes AP-1μ, which serves as an additional control. Panels separated by a dotted line represent different parts of the same blot. (D, E) HeLa cells were co-transfected with HIV-1 (D) or HIV-2 (E) proviral plasmids and no siRNA, siRNAs against AP-1γ, AP-2μ or AP-3δ, or a negative control siRNA (-ve), and observed by confocal immunofluorescence microscopy. Quantification of the effect of siRNAs on Gag distribution is shown graphically (D and E). For each siRNA treatment, at least 200 cells were assessed from three independent experiments by two different people. Cells were scored as having predominantly plasma membrane (PM), punctate intracellular (punctate), or diffuse cytoplasmic (diffuse) distribution of Gag. Error bars represent the standard deviation and significant changes from no siRNA treatment are shown with an asterisk (p < 0.05).

HIV-1 Gag has previously been reported to interact with the clathrin adaptor proteins AP-1 [16], AP-2 [17] and AP-3 [18]. AP-2 functions at the plasma membrane in clathrin-mediated endocytosis and has been shown to be inhibitory to HIV-1 particle release [17]. AP-1 and -3, involved in TGN-endosome and endosome-lysosome trafficking [19], respectively, are reported to be required for HIV-1 particle release. Furthermore, the distribution of HIV-1 Gag in cells derived from Hermansky-Pudlak syndrome patients, who lack functional AP-3, was shown to be different to that in wild type cells [26]. We used siRNA-mediated knock down to determine whether HIV-2 Gag interacts with the clathrin adaptor proteins in the same way. We have previously reported that prior siRNA knock down of AP-1 or AP-3 significantly affects the efficiency with which cells can be transfected with plasmid DNA [27]. Therefore, we co-transfected HeLa cells with siRNAs for AP-1, -2 or -3 at the same time as HIV-1 or HIV-2 proviral plasmids. Western blotting confirmed the efficient and specific knock down of AP-1γ, AP-2μ or AP-3δ subunits (Fig 3A–3C) and thus the level of the functional tetrameric complexes. First we tested whether knocking down AP-1, -2 or -3 had an effect on the distribution of HIV-1 and HIV-2 Gag by confocal immunofluorescence microscopy (Fig 3D and 3E respectively), which was quantified as described above. The presence of AP-2 siRNA or the negative control siRNA (-ve) had no significant effect on the distribution of HIV-1 Gag (Fig 3D). AP-1 siRNA significantly reduced the percentage of cells with plasma membrane-associated HIV-1 Gag, with corresponding small but insignificant increases in punctate and diffuse Gag. AP-3 siRNA also significantly reduced plasma membrane-associated Gag, with a corresponding increase in punctate Gag, a result that corroborates that seen with HIV-1 Gag in Hermansky-Pudlak syndrome cells [26]. For HIV-2 (Fig 3E), as with HIV-1 Gag, the presence of AP-2 or—ve siRNA had no significant effect on the distribution of HIV-2 Gag. AP-1 siRNA reduced the percentage of cells with plasma membrane-associated HIV-2 Gag and increased the percentage with punctate Gag. AP-3 siRNA had a greater effect, almost completely eliminating plasma membrane-associated Gag (2% of cells), with a corresponding increase in punctate Gag (98% of cells). These results demonstrate a similar role for AP-1 and AP-3 in allowing accumulation of HIV-2 Gag at the plasma membrane, and suggest that AP-3 at least may be required for trafficking of Gag from intracellular compartments to the plasma membrane. However, the fact that AP siRNA treatment had little effect on the majority of cells with a diffuse distribution of HIV-1 Gag suggests that the major trafficking pathways may be different for HIV-1 and HIV-2 Gag.

We next tested whether knock down of AP-1, -2 or -3 affected the levels of cell-associated and virion-associated full-length and processed Gag. During and after assembly of Gag and GagPol into particles, the viral protease cleaves Gag into its constituent proteins: MA, CA, NC, p6 (and two spacer peptides), which are then rearranged to produce the mature virion. Western blotting of cell lysates using anti-CA antibodies detects full length Gag (p55 and p57 for HIV-1 and -2 respectively), fully processed CA (p24 and p27 for HIV-1 and -2 respectively), and in the case of HIV-1 only, an MA-CA intermediate (p41). Western blotting of viral supernatants detects only fully processed p24 or p27 CA.

**Fig 4:**
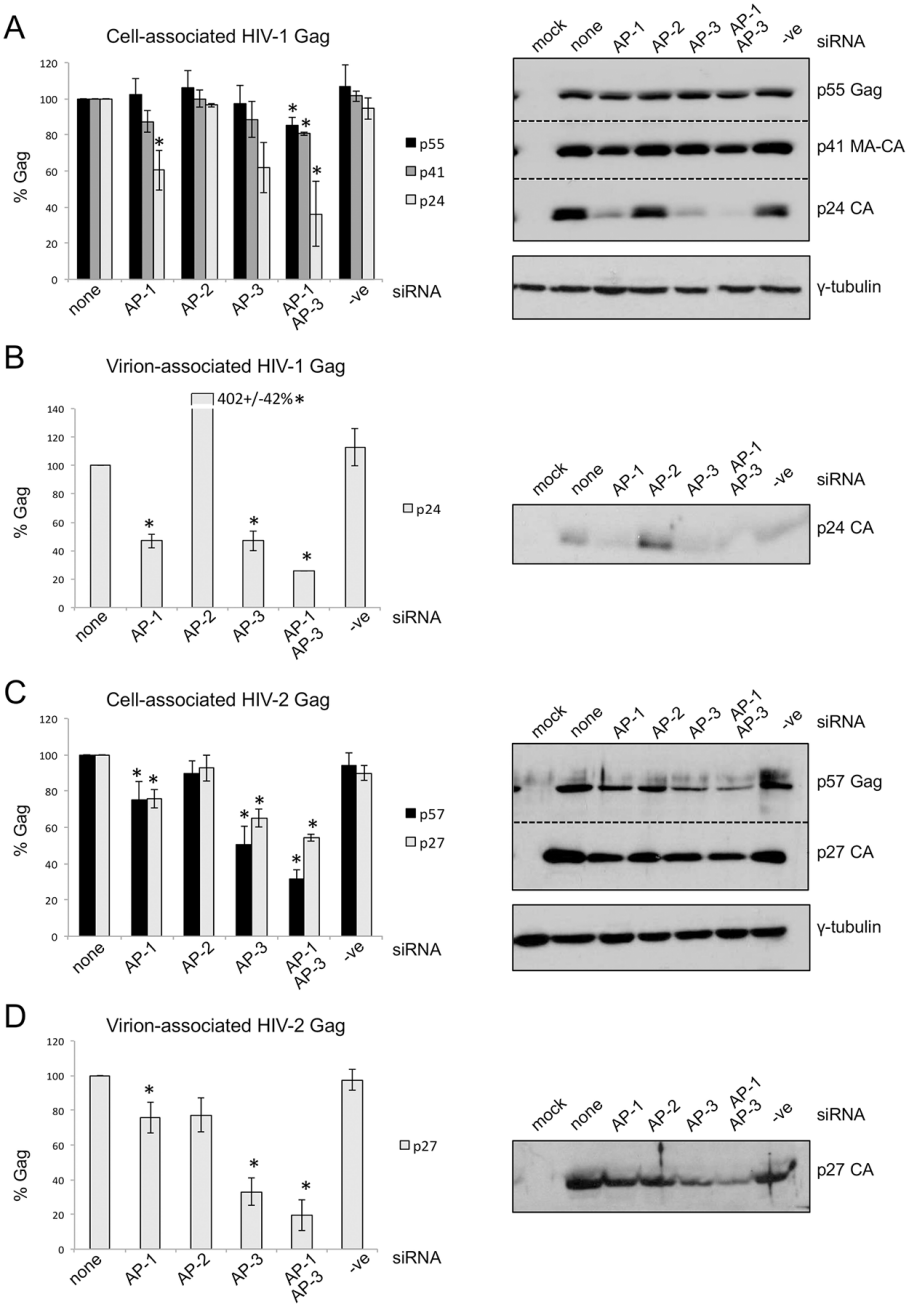
siRNA-mediated knockdown of AP-1, -2 and -3 have different effects on HIV-1 and HIV-2 particle release. HeLa cells were co-transfected with HIV-1 (A, B) or HIV-2 (C, D) proviral plasmids and no siRNA (none), siRNAs against AP-1γ, AP-2μ or AP-3δ, or a negative control siRNA (-ve). 42 hours post-transfection, cell lysates and viral supernatants were harvested. Cell-associated (A, C) and virion-associated (B, D) HIV-1 p55 (full length Gag), p41 (MA-CA) and p24 (CA), and HIV-2 p57 (full length Gag) and p27 (CA) were detected by western blotting (representative blots shown to the right) and quantified using densitometry. Error bars represent the standard error of the mean from three independent experiments and significant changes from no siRNA treatment are shown with an asterisk (p < 0.05).

For HIV-1 (Fig 4A and 4B), in agreement with previously published data [17], we found that AP-2 siRNA had no effect on cell-associated Gag, but greatly increased the amount of virion-associated p24 (CA). AP-1 or AP-3 siRNAs did not reduce the level of cell-associated p55 Gag, but reduced the level of virion-associated p24 (CA) by around 50%, as has been published previously [16, 18]. We did see a reduction in the relative level of p24 in cells treated with AP-1 siRNA (Fig 4A), suggesting either that Gag processing was reduced, (perhaps because less Gag was reaching the plasma membrane to assemble into particles), or that p24 was being degraded. We also treated cells with siRNAs for both AP-1 and AP-3 together. In this case, a small but significant reduction was seen in the level of cell-associated p55 Gag and p41 (MA-CA processing intermediate) and a greater reduction in cell-associated p24 (Fig 4A). There was also an additive effect of AP-1 and AP-3 siRNAs on virion release (Fig 4B), suggesting that AP-1 and AP-3 act on different trafficking pathways rather than the same one, although there may be some overlap. The results for HIV-2 (Fig 4C and 4D) were different. Firstly, knock down of AP-2 had no effect on cell- or virion-associated Gag, which shows that AP-2 is not inhibitory to HIV-2 particle release as it is for HIV-1. This suggests that HIV-2 Gag is not internalized from the plasma membrane by clathrin-mediated endocytosis. Secondly, AP-1 or AP-3 siRNA treatment reduced cell-associated p57 Gag by 25% and 50% respectively, with the level of cell-associated p27 (CA) reduced by a similar amount or less (25% and 35% for AP-1 and AP-3 siRNAs respectively; Fig 4C). This shows that AP-1 and particularly AP-3 siRNA treatment results in increased degradation of HIV-2 Gag, but also that HIV-2 Gag processing (and thus particle assembly) is not compromised by AP knock down. Thirdly, the level of virion-associated p27 was reduced by 25% in cells treated with AP-1 siRNA, suggesting that AP-1 siRNA had no further effect on virion release when compared with the reduction in cell-associated Gag, whereas AP-3 siRNA treatment reduced virion-associated p27 by 67% (Fig 4D), suggesting a further effect on release above the effect on cellular Gag levels. Again, treatment with AP-1 and AP-3 siRNAs together had an additive effect on both cell-associated p57 and p27, and virion-associated p27. Together these results suggest that AP-3 is required for the delivery or recycling of Gag and/or HIV-2 particles from intracellular sites to the extracellular space, and that accumulation of particles at these intracellular sites leads to their degradation.

### AP-3 and clathrin co-localize strongly with HIV-2 Gag

**Fig 5:**
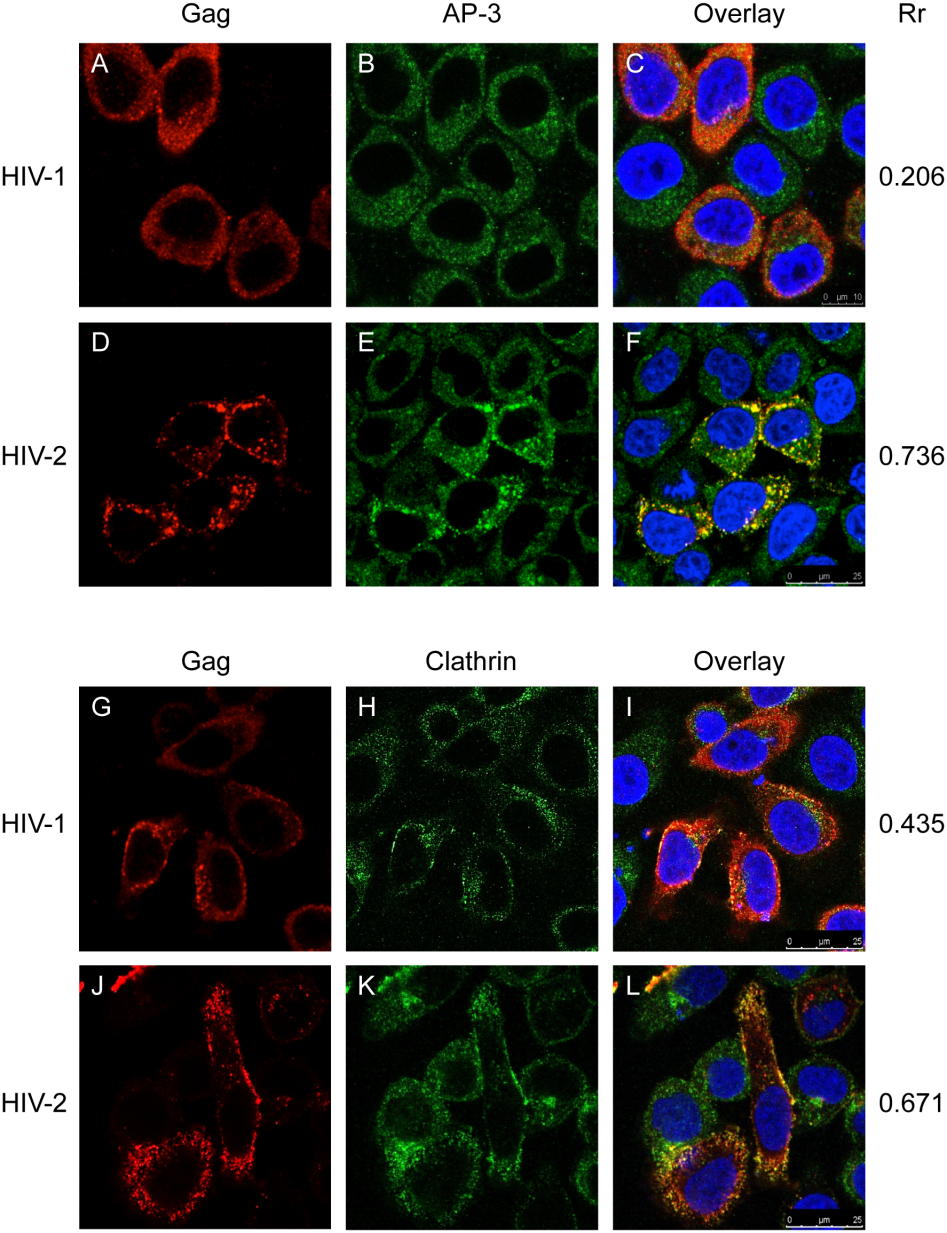
HIV-2 Gag co-localizes strongly with AP-3 and clathrin. Confocal immunofluorescence microscopy was carried out on HeLa cells transfected with HIV-1 (A-C, G-I) or HIV-2 (D-F, J-L) proviral plasmids. Gag is shown in red, AP-3 (B, C, E, F) or clathrin (H, I, K, L) are shown in green and DAPI is shown in blue. The overlay of the three channels is shown in the right hand image of each panel. Scale bars are shown in the bottom right hand corner of the overlay images. Mean Gag/AP-3 or Gag/clathrin co-localization (Pearson’s correlation coefficient, Rr) is shown to the right of each panel.

Given the possibility that AP-3 may function in the trafficking of HIV-2 Gag between intracellular compartments and the cell surface, we next looked at whether AP-3 co-localized with HIV-1 or HIV-2 Gag. Fig 5A–5C shows that HIV-1 Gag and AP-3 do not co-localize (Rr = 0.206) in HeLa cells whereas HIV-2 Gag and AP-3 co-localize strongly (Fig 5D–5F, Rr = 0.736). Furthermore, the distribution of AP-3 was different in cells expressing HIV-2 Gag, compared to untransfected cells, or those expressing HIV-1 Gag, as AP-3 appeared to be recruited to HIV-2 Gag-containing compartments. We also found this to be the case in 293T cells (data not shown). HIV-1 GagPol, HIV-2 Gag and SIVmac Gag have also been reported to interact with clathrin [28, 29]. AP-1 and AP-2 function in a primarily clathrin-dependent manner, while AP-3 has been reported to function both with clathrin and independently of it [30]. We therefore also looked at whether clathrin co-localized with HIV-1 or HIV-2 Gag. Fig 5G–5I shows that HIV-1 Gag and clathrin co-localize moderately (Rr = 0.435) in HeLa cells and HIV-2 Gag and clathrin co-localize strongly, both at the plasma membrane and within the cytoplasm (Rr = 0.671). Again, we saw the same result in 293T cells (data not shown). These results suggest that AP-3 and clathrin may be involved in the trafficking of HIV-2 Gag to/from intracellular compartments.

### A novel adaptor protein complex, AP-5, is involved in HIV-2 Gag trafficking

**Fig 6:**
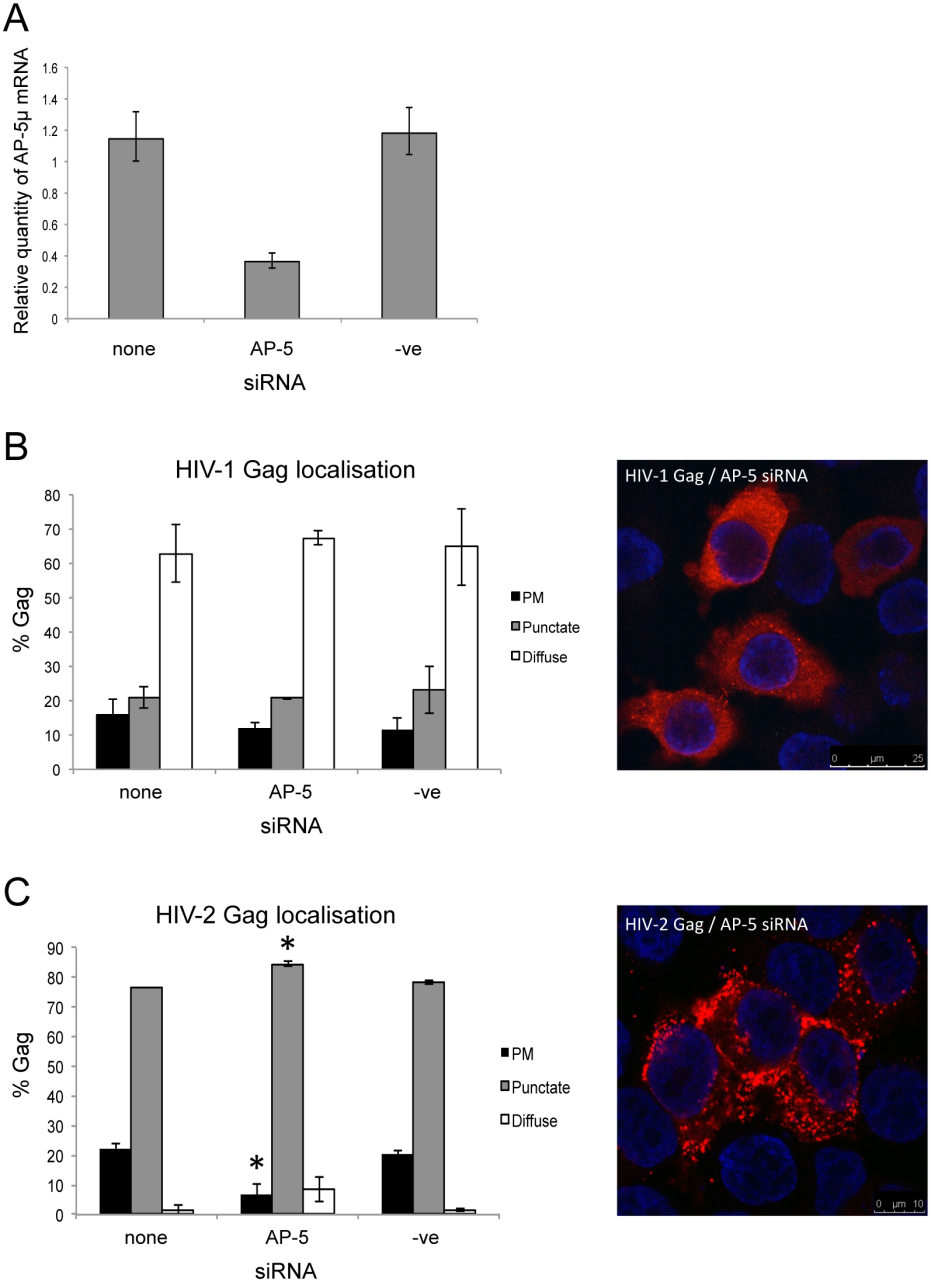
siRNA-mediated knockdown of AP-5 reduces plasma membrane localization and increases punctate distribution of HIV-2 Gag. (A) HeLa cells were transfected with 25 nM AP-5 siRNA or negative control siRNA (-ve), or mock transfected (none). Cells were harvested 42 hours post-transfection, from which RNA was isolated and RT-qPCR carried out to detect AP-5μ mRNA. Quantification was determined relative to GAPDH mRNA. (B, C) HeLa cells were co-transfected with HIV-1 (B) or HIV-2 (C) proviral plasmids and no siRNA (none), siRNA against AP-5μ or a negative control siRNA (-ve). Quantification of the effect of siRNAs on Gag distribution, as determined by confocal immunofluorescence microscopy, is shown graphically. For each siRNA treatment, at least 200 cells were assessed from three independent experiments. Cells were scored as having predominantly plasma membrane (PM), punctate intracellular (punctate), or diffuse cytoplasmic (diffuse) distribution of Gag. Representative confocal images are shown to the right, in which Gag is shown in red.

While carrying out this study, a new adaptor protein complex was discovered, namely AP-5 [20]. Interestingly, the UniProt entry for the mu5 subunit includes a reference to “putative HIV-1 interacting protein” although we were unable to find any published data to support this. We used an siRNA against AP-5μ previously reported [20] and tested its activity in HeLa cells by RT-qPCR since no effective antibody was available, achieving 65% knock down of AP-5μ at the mRNA level (Fig 6A). We then tested the effect of the AP-5 siRNA on HIV-1 and HIV-2 Gag localization in HeLa cells. Knock down of AP-5 had no effect on the distribution of HIV-1 Gag (Fig 6B), but it did reduce the percentage of cells with plasma membrane-associated HIV-2 Gag, with a corresponding increase in cells with punctate Gag distribution. An increase in the percentage of cells with diffuse distribution of Gag was also observed, although this change was not found to be significant. (Fig 6C).

**Fig 7:**
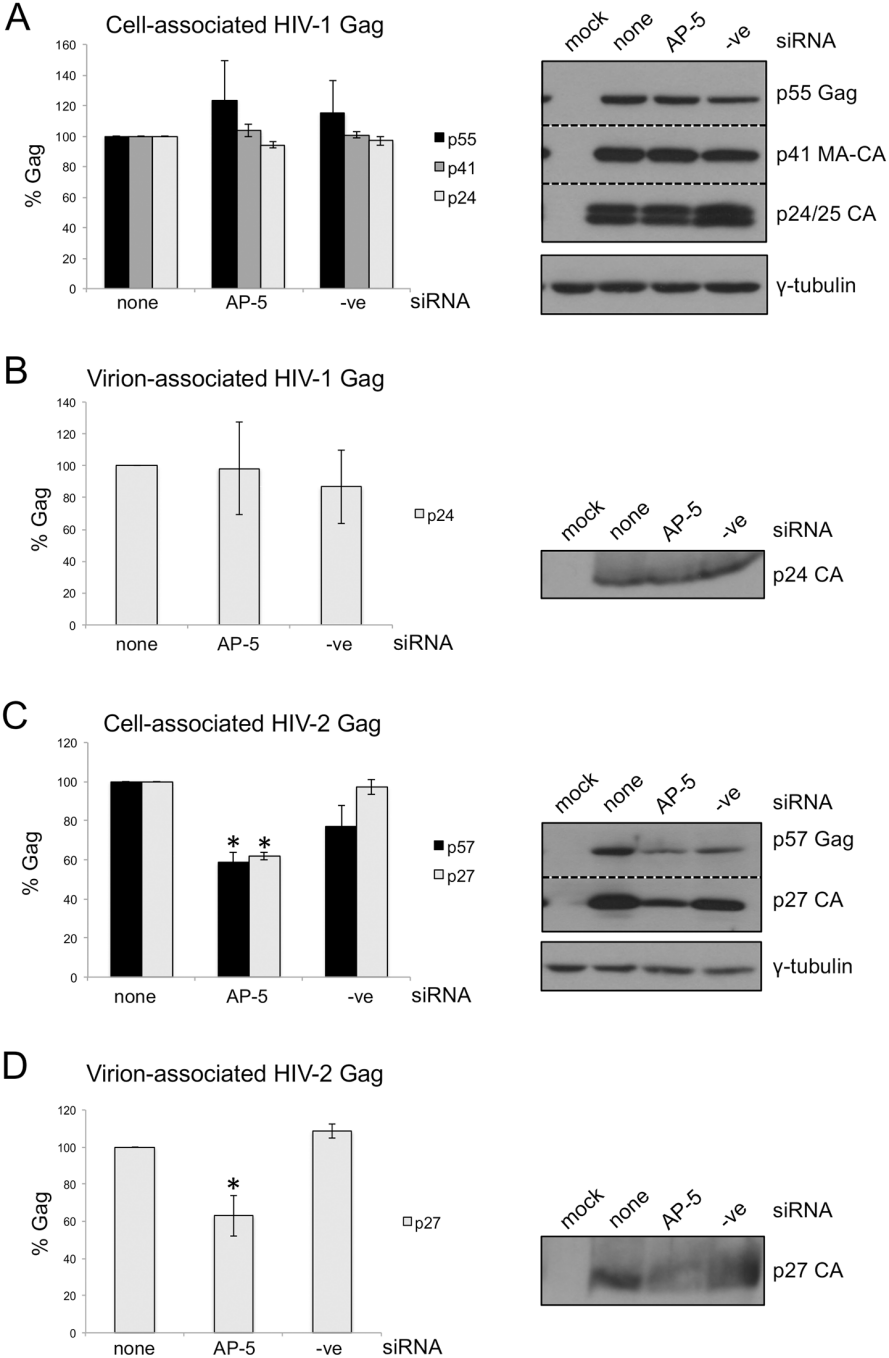
siRNA-mediated knockdown of AP-5 affects HIV-2 but not HIV-1 Gag. HeLa cells were co-transfected with HIV-1 (A, B) or HIV-2 (C, D) proviral plasmids and no siRNA (none), siRNA against AP-5μ or a negative control siRNA (-ve). 42 hours post-transfection, cell lysates and viral supernatants were harvested. Cell-associated (A, C) and virion-associated (B, D) HIV-1 p55 (full length Gag), p41 (MA-CA) and p24 (CA), and HIV-2 p57 (full length Gag) and p27 (CA) were detected by western blotting (representative blots shown to the right) and quantified using densitometry. Error bars represent the standard error of the mean from three independent experiments and significant changes from no siRNA treatment are shown with an asterisk (p < 0.05).

The relative levels of cell-associated and virion-associated HIV-1 and HIV-2 Gag were then measured. AP-5 siRNA had no effect on the levels of cell- or virion-associated HIV-1 Gag (Fig 7A and 7B respectively), but it reduced cell-associated HIV-2 p57 and p27 (Fig 7C), and virion-associated p27 (Fig 7D) by around 40%. This indicates that HIV-2 Gag is degraded in the absence of AP-5 but processing and release are not further affected. Since AP-5 has been shown to localize to late endosomal/lysosomal membranes [21], this suggests that AP-5 may be involved in retrieval of HIV-2 Gag and/or particles from late endosomes/lysosomes.

## Discussion

The data suggest that HIV-1 and HIV-2 Gag utilize different intracellular trafficking pathways to their sites of particle assembly and their release at the cell surface. HIV-1 Gag assembles at the plasma membrane in T cells, and in the cell lines used in this study; 293T and HeLa [8, 9]. As previously shown [16, 18], efficient HIV-1 particle release depends on the clathrin adaptor proteins AP-1 and AP-3, implicating vesicular transport of Gag to the plasma membrane. Gag binds to membranes via its myristylated N-terminal matrix (MA) domain [31], and therefore may be transported on vesicular membranes. The effect of siRNA-mediated inhibition of AP-1 or AP-3 expression suggests similar volumes of HIV-1 Gag trafficking via these two pathways. The increase in HIV-1 particle release when AP-2 is depleted (Fig 3, and previously shown [17]) confirms that a proportion of HIV-1 Gag is internalized from the plasma membrane by clathrin-mediated endocytosis. This work, and that of others [26] suggest that AP-1 and -3 may be involved in recycling HIV-1 Gag from endosomal compartments back to the cell surface.

In contrast to HIV-1, we have shown that intracellular compartments are major sites of HIV-2 Gag and/or particle accumulation in HeLa and 293T cells. Confocal immunofluorescence microscopy showed that HIV-2 Gag localized to CD63+/CD81+ puncta in 75% of cells. Changes in subcellular distribution of HIV-2 Gag, in levels of cell-associated Gag and extracellular virions, on siRNA-mediated depletion of AP-3 and -5, and strong co-localization of HIV-2 Gag with AP-3, were all suggestive of a role for late endosomal compartments in HIV-2 Gag trafficking. The data suggest that HIV-2 particles accumulate in late endosomal compartments and that particle release then depends on AP-3 and -5 (and to a much lesser extent AP-1) mediated recycling of late endosomal contents to the cell surface. If either of these adaptor proteins are depleted, a greater proportion of Gag undergoes lysosomal degradation. However, there are at least two possible interpretations of the route HIV-2 Gag takes to these compartments: 1) As HIV-2 Gag assembles at the plasma membrane, many of the assembling particles are internalized into endosomes. If this is the case, internalization occurs on a much greater scale than for HIV-1 and by a mechanism other than clathrin-mediated endocytosis, since depletion of AP-2 had no effect on HIV-2 particle release. 2) HIV-2 Gag assembles at intracellular membranes and buds into these compartments directly. Further work will be required to test these alternative, although not mutually exclusive, hypotheses.

Previous work has shown mechanistic differences between HIV-1 and -2 Gag in their membrane-binding, multimerization, and RNA-binding activities, which all affect particle assembly [32–34]. While recombinant HIV-2 Gag was shown to bind to the plasma membrane in yeast spheroplasts, it dissociated from the membrane more easily than HIV-1 Gag and did not form higher order multimers, resulting in a lack of virus-like particle production. However, these differences were not observed in human cell lines [32]. Structural studies of HIV-2 MA showed that the N-terminal myristate group, which is required for efficient membrane binding of Gag, is more tightly sequestered in its hydrophobic pocket in HIV-2 MA than HIV-1 MA, and does not easily switch to an exposed conformation, even on binding to PI(4,5)P_2_, the plasma membrane phosphoinositide that favours HIV-1 myristate exposure and Gag membrane binding [33]. Despite this, electron microscopy of HeLa cells transfected with HIV-2 proviral plasmid showed numerous extracellular viral particles in the vicinity plasma membrane, showing that viral particles could be assembled and released [33]. Recently, the RNA-binding and nucleic acid chaperone activities of HIV-1 and -2 Gag were shown to differ, with MA contributing to the chaperone activity of HIV-2 but not HIV-1 Gag [34]. These studies suggest that HIV-1 and HIV-2 have evolved slightly different strategies for particle assembly and release.

Interestingly, the newly identified adaptor protein complex AP-5, which localizes to late endosomal/lysosomal membranes [21], was found to be required for HIV-2, but not HIV-1, particle production. Of note, it is estimated that there are around 30-fold less copies of the AP-5 complex than either the AP-1 or AP-3 complex in HeLa cells [35]. The fact that siRNA knockdown of AP-5 had a significant effect on the level of cellular HIV-2 Gag despite the continued presence of a large excess of AP-1 and AP-3 suggests that it plays an important role in HIV-2 particle production.

The different pathways discussed all likely depend on the microtubule-dependent movement of vesicles or endosomal compartments. Movement along microtubules toward the cell periphery is usually driven by the plus-directed kinesin motor proteins [36]. HIV-1 Gag trafficking has been reported to require the kinesin KIF4 in COS-1 cells [37], and KIF3A in primary macrophages, where it was required for release of HIV-1 particles from VCCs [38]. AP-3 has been reported to interact directly with KIF3A [39], making this motor protein a good candidate for further studies on HIV-2 particle release.

In conclusion, the data presented in this study highlight that, in contrast to HIV-1, HIV-2 Gag accumulates in intracellular compartments in non-macrophage cells. HIV-2 particle production is dependent on the adaptor proteins AP-3 and AP-5 for delivery of particles from intracellular compartments to the cell surface, and their depletion leads to viral particle degradation. The implications of this research are that mechanistic differences between HIV-1 and HIV-2 in the trafficking, assembly and release of viral particles could contribute to the lower viral load observed during HIV-2 infection.
